# Brain and cognitive correlates of subjective cognitive decline-plus features in a population-based cohort

**DOI:** 10.1186/s13195-018-0449-9

**Published:** 2018-12-20

**Authors:** Gonzalo Sánchez-Benavides, Oriol Grau-Rivera, Marc Suárez-Calvet, Carolina Minguillon, Raffaele Cacciaglia, Nina Gramunt, Carles Falcon, ALFA Study, Jordi Camí, Grégory Operto, Stavros Skouras, Karine Fauria, Anna Brugulat-Serrat, Gemma Salvadó, Albina Polo, Laia Tenas, Paula Marne, Xavi Gotsens, Tania Menchón, Anna Soteras, Laura Hernandez, Ruth Dominguez, Sandra Pradas, Maria Pascual, Paula Marne, Maria León, Gema Huesa, Marc Vilanova, Sabrina Segundo, Jordi Huguet, Aleix Sala-Vila, Juan Domingo Gispert, José Luis Molinuevo

**Affiliations:** 1 Barcelonaβeta Brain Research Center, Pasqual Maragall Foundation, C/ Wellington 30, 08005 Barcelona, Spain; 2 CIBER Fragilidad y Envejecimiento Saludable (CIBERFES), Madrid, Spain; 3 Centro de Investigación Biomédica en Red de Bioingeniería, Biomateriales y Nanomedicina (CIBER-BBN), Madrid, Spain; 4 Universitat Pompeu Fabra, Barcelona, Spain

**Keywords:** Subjective cognitive decline, Memory, Voxel-based morphometry

## Abstract

**Background:**

Subjective cognitive decline (SCD) consists of self-perceived decline in cognition over time. The occurrence of specific additional features in SCD (so-called SCDplus) confers a higher risk of future cognitive decline. However, it is not known whether SCDplus patients have a distinct cognitive and neuroimaging profile. Therefore, we aimed to study the associations between SCDplus features and cognitive and neuroimaging profiles in a population-based cohort.

**Methods:**

A total of 2670 individuals from the ALFA cohort underwent clinical, cognitive, and MRI (*n* = 532) explorations. Subjects were classified as self-reporting cognitive decline (SCD) or not self-reporting cognitive decline (non-SCD). Within the SCD group, participants were also classified according to the number of SCDplus features they met (SCD+, > 3; SCD–, ≤ 3).

**Results:**

The prevalence of SCD in the cohort was 21.4% (55.8% SCD–, 44.2% SCD+). SCD+ subjects performed worse than non-SCD and SCD– subjects in memory and executive function. Among the SCDplus features, confirmation of decline by an informant was the best predictor of worse cognitive performance and lower gray matter volumes.

**Conclusions:**

Our findings show that individuals with SCDplus features have a distinct cognitive and brain volumetric profile similar to that found in Alzheimer’s disease and therefore support the use of the SCDplus concept as an enrichment criterion in population-based cohorts.

**Electronic supplementary material:**

The online version of this article (10.1186/s13195-018-0449-9) contains supplementary material, which is available to authorized users.

## Introduction

Subjective cognitive decline (SCD) has been suggested to be an initial manifestation of brain changes related to Alzheimer’s disease (AD) pathology [1]. SCD has been defined as *a self-experienced persistent decline in cognitive capacity in comparison with a previously normal status and not related to an acute event* [1, 2] and is supposed to precede mild cognitive impairment (MCI), characterized by objective cognitive impairment. Objective cognitive performance in SCD is by definition within normal ranges. The use of standardized tests has been shown to have limited ability to capture differences in performance between groups with and without SCD [3–8]. However, new approaches using more challenging tasks have found subtle deficits in the SCD group [9–12]. Furthermore, the presence of SCD increases the risk of cognitive decline and dementia [7, 13–17] and has been associated with lower volume in the medial temporal lobe, including the hippocampus [18–23], and other AD-related cortical areas [24–26] as compared to cognitively healthy subjects without SCD. Taken together, all of this latter evidence shows that some SCD subjects present structural changes that support the idea of SCD as the first clinical manifestation of AD.

Nevertheless, SCD is a complex syndrome that may be caused by multiple factors besides AD pathology, including other neurological or medical conditions, drug use, or psychological factors (see [27] for a review). Recently, the SCD-Initiative has proposed a set of specific SCD features, under the name SCDplus, which are associated with an increased likelihood to be an expression of the preclinical stage of AD [1, 2]. These features are: subjective decline in memory rather than other cognitive domains; onset in the last 5 years; age at onset > 60 years; concerns (worries) associated with SCD; feeling of worse performance than others of the same age group; confirmation of cognitive decline by an informant; and presence of the *APOE ε4* genotype. There is increasing interest in the study of individuals meeting SCDplus features to assess the usefulness of the SCDplus concept for enriching samples at higher risk of cognitive decline in AD secondary prevention trials. However, there is limited knowledge on which of the seven proposed features best relates to objective measures of cognition and/or cerebral changes. Most of the studies on SCD have so far included participants attending memory clinics. Recent evidence points out that the clinical, cognitive, and demographic characteristics [28, 29] and also the incidence of MCI [30] are different between clinical and population-based SCD samples. The identification of SCDplus cognitive and brain correlates in a population-based cohort would add to the understanding and usefulness of such a concept beyond memory clinics.

In this context, we hypothesize that some differences may be found in SCD subjects from a population-based cohort depending on the number of SCDplus features that they meet. Therefore, the aim of this study is to describe the cognitive and neuroimaging correlates of SCDplus. For this purpose, we describe the cognitive performance in persons with SCD after having classified them as a function of the number of SCDplus features they meet (≤ 3 and > 3); we investigate whether these features are related to cognitive performance or brain volumes in AD vulnerable areas (hippocampus); and, finally, we explore brain structural patterns related to the presence of SCDplus features and differences amongst non-SCD and SCD groups using an unbiased voxel-based morphometry (VBM) approach.

## Methods

### Participants

Participants of this study were assessed in the framework of the ALFA project (ClinicalTrials.gov, NCT01835717). ALFA participants (*n* = 2743) are cognitively healthy men and women aged between 45 and 74 years, most of them first-degree descendants of AD patients (47.4% with parental history of AD with onset before age 75 years). Exclusion criteria included scores below the cutoff values in cognitive screening tests (MMSE < 26, MIS < 6, semantic fluency < 12), CDR > 0, and major psychiatric disorders or diseases that could affect cognitive performance. The complete details on the ALFA study procedures and recruitment have been described in depth elsewhere [31]. For the present study, a total of 2670 subjects have been included since we excluded those individuals from which the *APOE* genotype was not available (*n* = 73). The study was approved by the Ethics Committee of the “Parc de Salut Mar” (Barcelona, Spain) and conducted in accordance with the directives of the Spanish Law 14/2007, of 3rd of July, on Biomedical Research. All participants signed an informed consent form and had a close relative, who also granted their consent, volunteering to participate in the study to give information of the participant’s cognitive and functional status.

### Assessment of subjective cognitive decline and classification of participants

Participants were classified as SCD if the answer to the question “Do you perceive memory or cognitive difficulties?” was affirmative. To collect standardized information on the perception of cognitive decline we used the Subjective Cognitive Decline Questionnaire (SCD-Q). The SCD-Q was devised to quantify the perceived subjective cognitive decline over the last 2 years and inquiries about the presence or absence of difficulties in 24 cognitive-related activities. The same set of questions is administered to both the subject (SCD-Q MyCog) and the informant (SCD-Q TheirCog) separately [32]. In addition, the presence or absence of six of the seven proposed SCDplus features was determined using either the information gathered by questions in the SCD-Q as proxies (i.e., concern focused in memory, worry about the decline, change in the last 2 years, and confirmation by an informant), sociodemographic data (i.e., age ≥ 60 years), or genetic testing (i.e., presence of at least one *APOE* ε4 allele). Informant confirmation of decline was determined based on the informant answer to the question “Do you perceive he/she has memory or cognitive difficulties?”. The SCDplus feature regarding the perception of worse performance than others in the same age group was omitted in the present study, as this information was not collected. The SCD group was dichotomized into two subgroups as a function of the number of SCDplus features that each participant met. The number of features was computed and participants were classified either as SCD minus (SCD*–*) if they presented ≤ 3 or as SCD plus (SCD+) if they presented > 3. Participants who did not report SCD (non-SCD) were used as the control sample.

### Neuropsychological measures

The battery used in this study includes measures of verbal episodic memory, executive function and working memory, visual processing, and verbal and nonverbal reasoning. Episodic memory was assessed with the Memory Binding Test (MBT), a word list-learning test devised to improve the detection of the earliest memory changes suggestive of AD [33]. The MBT encompasses the sequential learning of two sets of 16 words that share semantic categories. Free, cued, and paired recall are assessed in immediate and delayed (after 25–35 min) trials. We used the Spanish version of the MBT that we have recently adapted, normed, and validated [34]. The following main variables were gathered and analyzed: immediate total paired recall (TPR), immediate total free recall (TFR), total delayed paired recall (TDPR), and total delayed free recall (TDFR). The Wechsler IV Coding, Digit Span, Visual Puzzles, Similarities, and Matrix Reasoning subtests were also administered [35]. The coding task measures, among others, processing speed and attention. The Digit Span subtest evaluates short-term and working memory. Visual Puzzles measures visual reasoning and processing. Matrix Reasoning assesses fluid intelligence, and Similarities measures verbal reasoning and abstract thinking.

### Anxiety and depression

The Goldberg Anxiety and Depression Scale (GADS) was administered to assess the presence of anxiety and depressive symptoms. The scale is composed of two subscales with a maximum score of 9 points each. A global score is computed by summing up both subscales [36].

### MRI acquisition and preprocessing

A subgroup of 532 participants underwent a structural magnetic resonance imaging (MRI) study. This subgroup was selected as a function of their *APOE* genotype, aiming to maximize the number of *APOE ε4* allele carriers, which is known to be the most relevant genetic risk factor for AD [37]. Three-dimensional high-resolution T1-weighted images were obtained using a 3 T General Electric Discovery scanner with the following acquisition parameters: fast spoiled gradient-echo sequence, voxel size = 1 mm^3^ isotropic, repetition time (TR) = 6.16 ms, echo time (TE) = 2.33 ms, inversion time (TI) = 450 ms, matrix size = 256 × 256 × 174, flip angle = 12°. Images were processed to perform voxel-wise statistics with the following procedure. They were segmented into gray matter (GM) tissue using the new segment function implemented in Statistical Parametrical Mapping software (SPM 12; Wellcome Department of Imaging Neuroscience, London, UK). The DARTEL toolbox was used to generate a reference template object of the sample which was warped into a standard MNI space. The generated flow fields and normalization parameters were then implemented to normalize the native GM T1 images to the MNI space. Jacobian determinants were applied to preserve the local native amount of gray matter (modulated images). Finally, images were spatially smoothed with a 10-mm full-width at half maximum (FWHM) Gaussian kernel. Additionally, the total intracranial volume (TIV) was computed by summing the segmented GM, WM, and CSF for each individual. Hippocampal volumes were automatically calculated by FreeSurfer (v5.3; surfer.nmr.mgh.harvard.edu) using whole hippocampal segmentation provided by the standard ‘recon-all’ pipeline, which renders the most standard and widely-used volumetric estimation of hippocampal volumes. Additional details on the computation of hippocampal volumes by this pipeline can be found in [38]. Raw hippocampal volumes were adjusted by dividing them by the TIV.

### Statistical analyses

We compared the means in sociodemographic and cognitive screening variables as well as *APOE ε4* allele frequency distributions between non-SCD subjects and the whole SCD group by means of *t* tests for continuous variables and chi-square tests for categorical variables. After splitting the SCD group into two subgroups as a function of the number of SCDplus features they meet (SCD– and SCD+), these variables were compared by one-way ANOVA and pairwise post-hoc tests (Tukey) and chi-square tests. Similarly, cognitive performance was firstly compared between non-SCD subjects and the SCD group as a whole and, secondly, among non-SCD subjects and SCD subgroups using ANCOVAs (with age, years of education, sex, number of *APOE ε4* alleles, and mood scores as covariates). In this second analysis, both the main effect of group (non-SCD/SCD–/SCD+) and post-hoc pairwise differences were tested. TIV-adjusted hippocampal volumes were first compared among SCD groups by mean ANOVAs and subsequently using covariation by age, sex, and number of *APOE ε4* alleles using ANCOVAs. In an additional analysis, the association between the independent SCDplus features and cognitive performance was tested with linear models in which SCDplus features were included as predictor variables, cognitive performance as dependent variables, and age, education, sex, and mood scores as covariates. Similarly, models with the TIV-adjusted hippocampal volume as the dependent variable were constructed to explore its association with the SCDplus features. Statistical analysis was performed in SPSS IBM v22. All tests were two-tailed, with a significant level of α = 0.05.

Voxel-wise analyses were performed using the general linear model as implemented in SPM 12. The segmented, modulated, and smoothed GM images were entered in a full-factorial design and differences in GM volume were explored using pairwise contrasts among the non-SCD, SCD–, and SCD+ groups. Age, education, sex, mood scores, and TIV were used as nuisance variables. In addition, we introduced the number of *APOE ε4* alleles as a covariate to regress its effect out of our VBM models under the assumption that the effect of the number of alleles is additive [39]. Additional models were constructed to explore the association between SCDplus features and GM volumes. Firstly, we tested the association between the number of SCDplus features met and GM volume using covariation by age, education, sex, and TIV. Secondly, a model was built including the six binary individual features as effects of interest and age, education, sex, and TIV as covariates. Contrasts for each feature were tested separately. An uncorrected *p* value (< 0.001) and a minimum cluster size (*k* = 100) were used as significance thresholds in all VBM analyses performed due to the exploratory nature of the study assuming that few differences between these groups of healthy participants would be observed. Family-wise error (FWE)-corrected *p* values were also computed and provided for reference.

## Results

### Prevalence of SCD and SCDplus features

Among the ALFA participants included (*n* = 2670), 21.4% (*n* = 572) accomplished criteria for SCD. With regard to SCDplus features, all reported memory as the main compromised domain, 72% experienced the decline within the last 2 years, 67.8% expressed worries about their cognition, 34.3% were *APOE ε4* carriers, 34.1% were older than 60 years, and, finally, 28% had confirmation of decline from an informant. Prevalence of features by SCD subgroups (SCD+ and SCD–) are presented in Table 1. See Additional file 1 for a more detailed description of frequency and co-occurrence of SCDplus features (Additional file 1: Tables S2 and S3 and Figures S1 and S2).

**Table 1:** Sociodemographic, genetic, mood, and SCDplus features descriptive data in the whole sample

	Non-SCD group	SCD– group	SCD+ group
*N*	2098	319	253
Age, mean (SD)	55.41 (6.62)	55.62 (6.22)	59.10 (7.12)*^,^**
Education, mean (SD)	13.41 (3.52)	13.38 (3.55)	12.83 (3.41)*
Females, *n* (%)	1316 (62.7%)	218 (68.3%)	154 (60.9%)
GADS—anxiety, mean (SD)	0.49 (1.07)	0.92 (1.51)*	0.90 (1.41)*
GADS—depression, mean (SD)	0.11 (0.52)	0.35 (0.93)*	0.44 (1.07)*
SCDplus features, *n* (%)			
Worry about the decline		165 (51.7%)	223 (88.1%)**
Change in the last 2 years		179 (56.1%)	233 (92.1%)**
Age > 60 years		62 (19.4%)	133 (52.6%)**
Confirmation by informant^a^		37 (11.6%)	122 (48.2%)**
*APOE ε4* carriers	731 (34.8%)	52 (16.3%)^*^	144 (56.9%)*^,^**

*SCDplus* subjective cognitive decline plus*, non-SCD* not self-reporting cognitive decline, *SCD–* SCD with ≤ 3 SCDplus features, *SCD+* SCD with >3 SCDplus features, *SCD* subjective cognitive decline, *SD* standard deviation, *GADS* Goldberg Anxiety and Depression Scale, *APOE* apolipoprotein E, *ANOVA* analysis of variance

^a^Data available in 568 out of 572 participants with SCD

^*^p < 0.05 as compared to non-SCD; pairwise (Tukey) ANOVA/chi-square test

^**^p < 0.05 as compared to SCD–; pairwise (Tukey) ANOVA/chi-square test

As a whole group, SCD individuals were older and reported higher anxiety and depressive symptoms than participants not presenting SCD (*p* < 0.001). Table 1 presents descriptive data for sociodemographic information, the number of *APOE ε4* carriers, and mood scores by SCD categories. As expected, SCD+ individuals were older and had a higher prevalence of the *APOE ε4* allele than the non-SCD and SCD– groups (*p* < 0.001). We did not find statistically significant differences in the anxiety and depression subscales of the GADS between the SCD+ and SCD– subgroups. Regarding SCD-Q scores, SCD+ subjects obtained higher scores than the SCD– and non-SCD subjects (*p* < 0.001). This held true for both the participant scores (MyCog) and the informant scores (TheirCog). Noteworthy, self-reported difficulties (assessed as the overall MyCog score) in all groups were higher than informant-derived reports (TheirCog overall score) with increasing values following the pattern non-SCD < SCD– < SCD+.

The subsample of subjects who underwent MRI was on average 1 year younger (*p* < 0.05) and slightly more educated (0.3 years of education, *p* < 0.05) than the group that was not scanned. Sociodemographic, genetic, and mood data of this subgroup are presented in Additional file 1: Table S1. The percentage of *APOE ε4* allele carriers is overrepresented in the MRI subsample (30.7% vs 51%, *p* < 0.001). No significant differences in mood or cognitive outcomes were found, except a marginally better performance of scanned individuals in the Similarities subtest (*p* < 0.05). With regard to SCD prevalence, it was similar in both groups (21.7% vs 20.5% in the MRI subsample, *p* < 0.54). Noticeably, the number of subjects classified as SCD+ was higher in the MRI subsample (44.2% vs 66.1%, *p* < 0.05).

### Cognitive outcomes

**Fig. 1:**
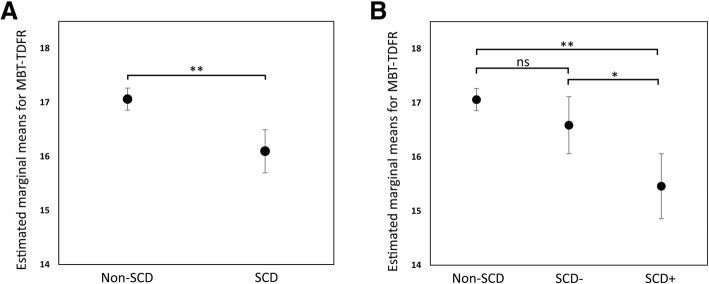
Estimated marginal means for total delayed free recall (TDFR) of the Memory Binding Test (MBT) by SCD group. **a** Non-SCD vs SCD. **b** Non-SCD vs SCD subgroups by number of SCDplus features met (SCD+, SCD-). * *p* < 0.005, ** *p* < 0.0001, ns not significant, SCD self-reporting cognitive decline, non-SCD not self-reporting cognitive decline, SCD+ SCD with > 3 subjective cognitive decline plus features, SCD– SCD with ≤3 subjective cognitive decline plus features

Participants with SCD obtained lower scores than non-SCD subjects in all memory variables (*p* < 0.05). However, we found that these differences were mainly driven by the performance of the SCD+ group, because this group presented statistically significant differences with respect to the non-SCD group, while the SCD– group did not (Table 2). The SCD+ group also displayed significantly lower scores than non-SCD participants in Coding. This difference survived a Bonferroni-type correction for multiple comparisons using a more restrictive *p* value (*p* < 0.005), as did the MBT-TDFR variable, in which the SCD+ group showed significant differences as compared to both the SCD– group and the non-SCD group. Figure 1 provides a graphic example for the MBT-TDFR. Although differences were statistically significant, the magnitudes of the effects were small (largest partial η^2^ = 0.009).

**Table 2:** SCD-Q and cognitive scores by SCD group

	Non-SCD group	SCD– group	SCD+ group
*N*	2098	319	253
SCD-Q MyCog	4.35 (4.06)	10.39 (5.66)*	14.04 (5.41)*^,^**
SCD-Q TheirCog	2.78 (2.44)	4.02 (4.03)*	6.39 (5.25)*^,^**
MBT-TPR	24.29 (4.26)	23.89 (4.48)	22.52 (4.98)*^,^**
MBT-TFR	16.74 (4.93)	16.36 (4.90)	14.51 (5.11)*^,^**
MBT-TDFR	17.14 (3.02)	16.68 (5.42)	14.67 (5.23)*^,^**
MBT-TDPR	24.11 (4.41)	23.75 (4.62)	22.23 (5.17)*^,^**
WAIS—Coding	66.67 (14.6)	65.63 (14.91)	59.64 (13.90)*
WAIS—Visual Puzzles	13.55 (4.30)	12.90 (4.12)	12.26 (3.90)
WAIS—Digit Span Total	24.93 (5.11)	24.66 (5.33)	23.86 (5.58)
WAIS—Matrix Reasoning	16.77 (4.35)	16.31 (4. 51)	15.41 (4.48)
WAIS—Similarities	22.34 (4.64)	21.67 (4.80)	21.74 (4.73)

Data presented as mean (standard deviation)

*SCD-Q* Subjective Cognitive Decline Questionnaire, *SCD* subjective cognitive decline, *non-SCD* not self-reporting cognitive decline, *SCD–* SCD with ≤ 3 SCDplus features, *SCD+* SCD with > 3 SCDplus features, *SCDplus* subjective cognitive decline plus*, MyCog* questions to participant, *TheirCog* questions to informant, *MBT* Memory Binding Test, *TPR* total paired recall, *TFR* total free recall, *TDFR* total delayed free recall, *TDPR* total delayed paired recall, *WAIS* Wechsler Adult Intelligence Scale, *APOE* apolipoprotein E

^*^p < 0.05 as compared to non-SCD

^**^p < 0.05 as compared to SCD–. Analysis of variance and post-hoc Tukey test for SCD-Q; pairwise (Least Significant Difference) analysis of covariance with age, education, sex, number of *APOE ε4* alleles, anxiety, and depression as covariates for cognitive outcomes

### Neuroimaging results for group comparisons

**Fig. 2:**
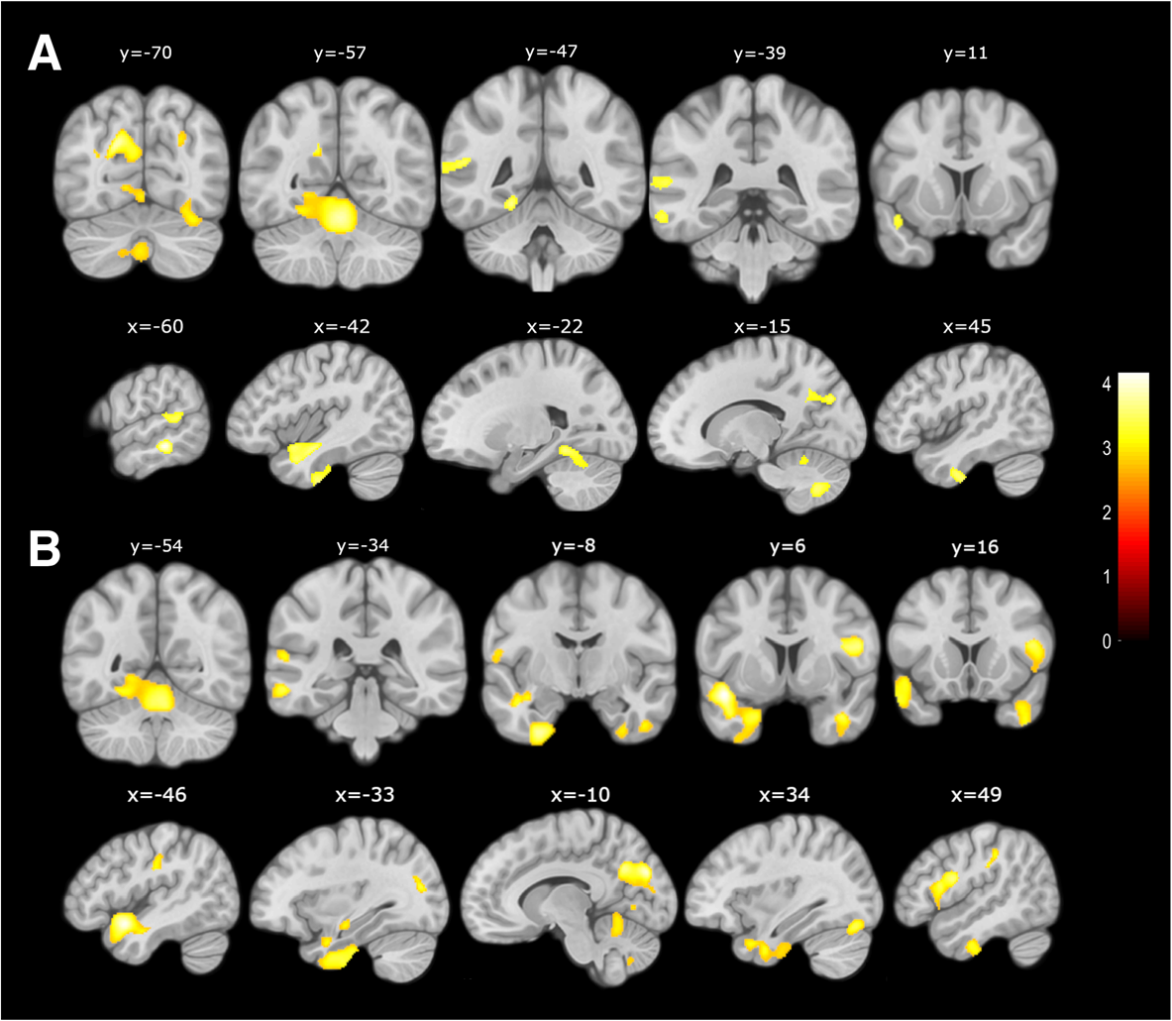
SPM maps showing contrasts which showed significant differences at *p* < 0.001 and minimum cluster size *k* = 100. **a** Areas of decreased gray matter volume of SCD+ subjects as compared to SCD– subjects (SCD– > SCD+). **b** Areas of increased gray matter volume of SCD– subjects as compared to non-SCD subjects (SCD– > non-SCD)

The SCD+ group showed lower left TIV-adjusted hippocampal volumes than the non-SCD (*p* = 0.004) and SCD– (*p* = 0.017) groups in unadjusted comparisons. However, after covariation by age, sex, and number of *APOE ε4* alleles this difference was no longer significant (*p* < 0.05). In the VBM analysis, the SCD+ group showed significantly lower GM volume than the SCD– group in regions known to be affected in AD such as the bilateral temporal cortices, fusiform and lingual gyri, precuneus and cuneus, and medial cerebellum (Fig. 2a and Table 3). No significant differences were detected between the SCD+ and non-SCD groups. On the other hand, the SCD– group showed increased GM volume with respect to the non-SCD group in a very similar pattern as observed in the comparison between the SCD– and SCD+ groups (Fig. 2b and Table 3).

**Table 3:** Brain regions showing statistically significant gray matter volumetric decrease in pairwise comparisons between non-SCD, SCD+, and SCD– groups

Contrast		Label	MNI coordinates			Cluster	Peak	pFWE-corr	
			*x*	*y*	*z*	size	*Z* score	Peak	Cluster
SCD– > SCD+	Cluster 1	Left temporal middle	−59	−33	−12	345	4.13	0.117	0.459
		Left temporal inferior							
	Cluster 2	Left temporal inferior	−33	−15	−44	801	4.05	0.151	0.164
		Left fusiform							
	Cluster 3	Left temporal pole superior	−38	−11	−17	753	3.81	0.315	0.182
		Left temporal middle							
		Left temporal superior							
		Left temporal pole middle							
	Cluster 4	Right cerebellum	30	−74	−20	734	3.73	0.393	0.190
		Right Fusiform							
	Cluster 5	Left postcentral	−53	−20	30	208	3.72	0.401	0.625
		Left supramarginal							
	Cluster 7	Right cerebellum	12	−66	−47	556	3.72	0.403	0.283
		Vermis							
	Cluster 8	Vermis	−2	−59	−18	1540	3.68	0.437	0.037
		Left cerebellum							
		Left fusiform							
		Right cerebellum							
	Cluster 9	Left cerebellum	−14	−68	−45	559	3.63	0.494	0.281
	Cluster 10	Left occipital superior	−17	−77	29	340	3.59	0.536	0.464
		Left precuneus							
		Left cuneus							
	Cluster 11	Right temporal inferior	47	−9	−38	420	3.59	0.541	0.386
		Right temporal pole							
		Right fusiform							
	Cluster 12	Left temporal superior	−54	−45	15	428	3.54	0.593	0.379
		Left temporal middle							
SCD– > non-SCD	Cluster 1	Left temporal pole superior	−45	6	−17	4502	4.66	0.015	0.000
		Left fusiform							
		Left temporal pole middle							
		Left temporal superior							
		Left hippocampus							
		Left parahippocampal							
		Left insula							
		Left amygdala							
	Cluster 2	Left cuneus	−17	−63	33	2216	4.56	0.022	0.011
		Left occipital superior							
		Left precuneus							
		Right cuneus							
		Left calcarine							
	Cluster 3	Right fusiform	30	−77	−17	956	4.31	0.061	0.118
		Right cerebellum							
		Right lingual							
		Right occipital inferior							
	Cluster 4	Left lingual	2	−57	−18	3020	4.28	0.068	0.003
		Vermis							
		Left cerebellum							
		Left fusiform							
	Cluster 5	Right temporal pole middle	39	9	−33	1723	4.15	0.109	0.026
		Right temporal inferior							
		Right temporal pole superior							
	Cluster 6	Right frontal inferior	50	2	20	1157	4.11	0.126	0.078
		Right rolandic							
		Right precentral							
	Cluster 7	Left temporal middle	−59	−33	−12	317	3.99	0.183	0.489
		Left temporal inferior							
	Cluster 8	Left occipital middle	−32	−75	15	123	3.93	0.220	0.749
	Cluster 9	Right supramarginal	45	−30	33	189	3.72	0.404	0.651
		Right postcentral							
	Cluster 10	Left temporal superior	−59	−35	12	250	3.58	0.551	0.569
		Left temporal middle							
	Cluster 11	Left postcentral	−53	−17	32	396	3.58	0.554	0.408
		Left parietal inferior							
		Left supramarginal							
	Cluster 12	Right cerebellum	−3	−68	−45	489	3.56	0.601	0.330
		Left cerebellum							
	Cluster 13	Left rolandic	−60	−3	12	229	3.53	0.624	0.596
		Left postcentral							
	Cluster 14	Right occipital superior	26	−71	33	109	3.40	0.617	0.771

*non-SCD* not self-reporting cognitive decline, *SCD–* SCD with ≤ 3 SCDplus features, *SCD+* SCD with > 3 SCDplus features, *SCDplus* subjective cognitive decline plus*, MNI* Montreal Neurological Institute, pFWE Family-wise Error-adjusted *p*-value

### Effect of SCDplus features in cognition and neuroimaging outcomes

We found that confirmation of cognitive decline by an informant predicted the MBT immediate and delayed total free recalls (TFR and TDFR) (TFR, standardized *β* = − 0.31, *p* = 0.046; TDFR, standardized *β* = − 0.37, *p* = 0.004). In addition, both confirmation by an informant and age > 60 years predicted performance in the Coding subtest (confirmation by informant, standardized *β* = − 0.24, *p* = 0.046; age > 60 years, standardized *β* = − 0.33, *p* = 0.005). Furthermore, we found a positive effect in the performance on Matrix Reasoning for being worried about the decline (standardized *β* = 0.17, *p* = 0.025). No other features significantly predicted performance in the cognitive outcomes assessed here. Setting a more restrictive *p* value to adjust for multiple comparisons (*p* > 0.005), the relationship between TDFR and confirmation of decline and the relationship between age > 60 years and Coding were still significant.

**Fig. 3:**
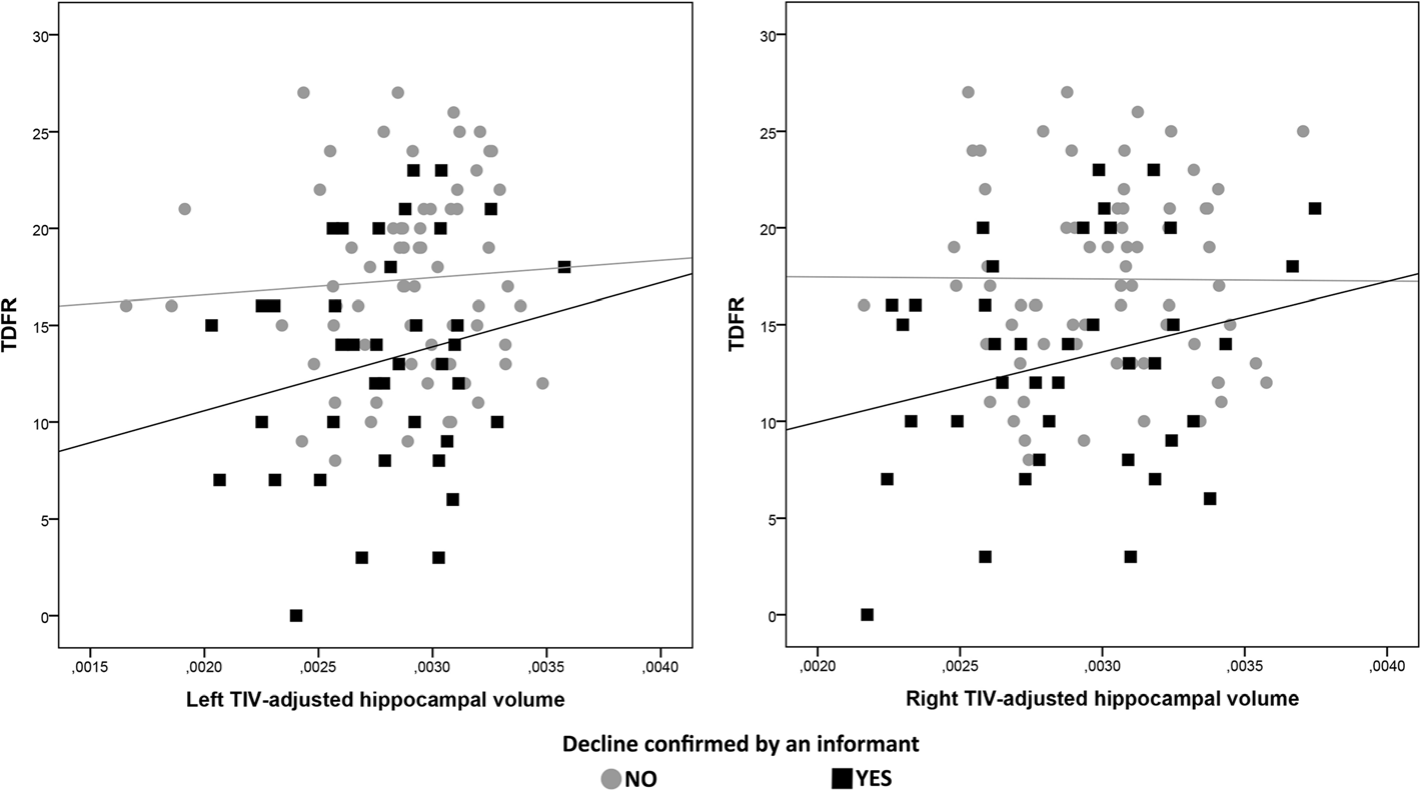
Association between memory scores and TIV-adjusted hippocampal volumes by presence/absence of informant confirmation of decline. TDFR total delayed free recall, TIV total intracraneal volume

**Fig. 4:**
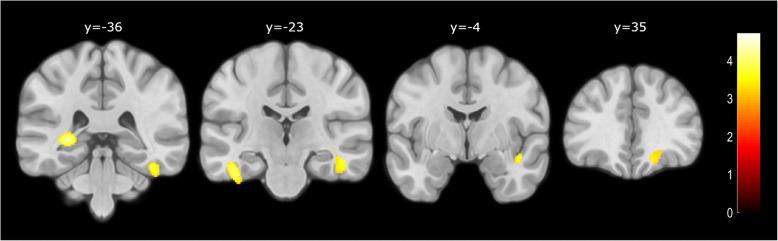
Areas in which confirmation of cognitive decline by an informant is associated with decreased gray matter volume

With regard to neuroimaging outcomes, confirmation of the cognitive decline by an informant was the only SCDplus feature that predicted lower hippocampal volume (left hippocampus, standardized *β* = − 0.20, *p* = 0.033; right hippocampus, standardized *β* = − 0.21, *p* = 0.046). In a posterior analysis, we explored the correlation between hippocampal volume and episodic memory performance (TFR and TDFR) accounting for the presence or absence of informant confirmation of decline. A positive correlation with TDFR was found in SCD subjects who had informant confirmation (*n* = 39), although no significance was reached (left hippocampus, *r* = 0.20, *p* = 0.22; right hippocampus, *r* = 0.25, *p* = 0.12; Fig. 3). This finding was confirmed by the results obtained from the VBM analysis. Confirmation of the decline by the informant was the feature that more consistently showed association with lower GM volume in AD-related areas, encompassing the bilateral inferior temporal lobe, left hippocampus, right insula, and right orbitofrontal cortex (Fig. 4). The SCDplus feature of being older than 60 years was associated with lower GM volume in the right supramarginal gyrus (> 60 years), while reporting worries about the decline was associated with lower GM in the right superior temporal gyrus and superior parietal, and left insula. We found a negative association between the number of SCDplus features met by SCD participants and GM local volumes showing a topographical distribution that included areas in the temporal lobe and precuneus bilaterally, the cerebellum, and some frontal regions (see Additional file 1: Figure S3 and Table S4).

## Discussion

In this study, performed in a large community-based sample of middle-aged healthy subjects, we aimed to determine whether meeting more than three SCDplus features had specific cognitive and neuroimaging correlates, and also to explore which features better predicted cognitive performance and GM volume. Overall, our findings support the use of the concept of SCDplus also in population-based cohorts since performance in subjective decliners was lower than that in non-SCD participants only in the individuals meeting more than three SCDplus features (SCD+ group). This group also showed a decrease of volume in AD-related brain areas as compared to the SCD group meeting fewer features. We found that confirmation of decline by an informant was the best cognitive and GM volume predictor.

In this population-based cohort, 572 out of the 2670 subjects studied reported SCD, representing a prevalence of 21.4%. SCD prevalence is highly variable among studies, depending upon sample recruitment strategies as well as the SCD definition [40], our findings being in accordance with previous community-dwelling SCD studies [41, 42]. As compared to non-SCD participants, subjects with SCD reported higher anxiety and depressive symptoms and had higher scores on the SCD-Q questionnaire. Besides the compromise in memory, the most prevalent SCDplus features were a reported decline in the last 2 years and the presence of worries about the decline.

We found a significant negative impact of SCD in cognition, mainly driven by the SCD+ group (individuals meeting > 3 SCDplus features). A significant lower performance in this group was observed as compared to both non-SCD subjects and subjects in the SCD– group, while no differences between these latter groups were found. These findings could not be attributable to confounders, since adjustments for demographics and mood scores were implemented in the analysis. Differences were focused in the episodic memory domain, which mainly relies on the integrity of medial temporal lobe regions [43]. There is a well-described spread of neuropathology from these regions to wide cortical areas in the course of symptomatic AD, symptoms being related to the topographical distribution of tau pathology. Recently, evidence of increased tau deposition has been reported in the medial temporal lobe of healthy elders with SCD [44], and our findings on the predominance of memory subclinical deficits in subjects with SCD may be related to such changes.

Our results for cognitive outcomes partially mirror the findings reported by Fernández-Blázquez et al. in subjects who fulfill SCDplus features [45]. They found lower scores in SCDplus as compared to non-SCD subjects only for verbal episodic memory measured with the Free and Cued Selective Reminding Test (FCSRT). Interestingly, in their study they found differences between SCDplus and SCD restricted to the delayed free recall scores. In our study we used the MBT, which is a test that has similar advantages to the FCSRT (i.e., controlled learning and distinction between storage and retrieval) but overcomes its limitations, such as the ceiling scores achieved by subjects with any or subtle memory deficits [46]. By using a larger sample and the MBT, we were able to demonstrate global memory differences, in both free and cued measures, between the SCD+ and SCD– groups.

We also found differences in the Coding subtest of the WAIS-IV. This kind of task, which measures attention and psychomotor and processing speed, is one of the most sensitive tools to detect cognitive impact due to any brain insult but lacks disease-related specificity [47]. Noteworthy, this test was included in the longitudinal data-derived preclinical Alzheimer cognitive composite (PACC) and, together with episodic memory and orientation measures, has shown good performance in detecting decline in cognitively healthy subjects with evidence of AD pathology [48].

With regard to the ability of specific SCDplus features to predict cognitive performance, in our study, confirmation of the decline by an informant was the feature that best related to objective cognitive performance in memory and executive function tasks. In addition, this feature was also the only one that predicted hippocampal volume and related to lower GM volume in AD-relevant regions. This is in line with several reports highlighting the value of informant ratings in SCD and preclinical AD in which informant complaints predicted progression to AD dementia in longitudinal studies [49, 50]. Recently, Valech et al. [51] demonstrated the superiority of informant-related ratings over self-reported ones when it comes to discriminating between controls and biomarker-defined preclinical AD subjects. In addition, they found that informant measures also correlated better with CSF AD biomarkers than self-derived measures. The rationale behind this phenomenon is proposed to be two-fold: first, some subjects in the AD preclinical stage may present some initial form of anosognosia (unawareness of the own difficulties); and second, a proportion of the cognitive decliners report subjective changes related to other conditions, such as psychoaffective symptoms, or personality traits, such as neuroticism. In this regard, in our study, subjects with SCD reported higher anxiety and depressive symptoms as compared to non-SCD subjects, as previously reported [28, 52]. This finding is in agreement with the attentional bias toward negative information that depressed individuals usually present [53], which would make them more sensitive to their cognitive failures [54] and points toward an overlap between the subjective perception of decline due to preclinical AD and due to mood-related causes. In both scenarios, anosognosia and mood-driven complaints, external reports appear as less biased indicators of the presence of actual subclinical decline. Investigators in the INSIGHT-preAD study have further explored this by developing an “awareness indicator”, operationalized as the difference between informant-reported and self-reported scores. This type of measure seems promising since, by classifying subjects in “low *versus* high awareness” groups, significant differences that were not captured by isolated SCD scores appear in amyloidosis and reduced cortical metabolism [4].

With regard to other SCDplus features, we found that being older than 60 years, even after age adjustment, predicted lower performance in Coding. This finding suggests higher executive/processing speed impact of SCD than that expected by age. In contrast, we found a positive relationship between having worries about the decline and Matrix Reasoning performance. An association between higher insight and good reasoning abilities could underlie this unexpected association.

As expected, the SCD+ group showed lower GM volume than the SCD– one in areas known to be affected by AD. On the other hand, we found that the non-SCD group showed no significant differences with respect to the SCD+ group, and also displayed lower GM volumes than the SCD– group. This effect overlapped with those regions with decreased GM volume in the SCD+ group as compared to the SCD– group. Indeed, in an independent population composed of subjects in the AD continuum from healthy controls to preclinical and symptomatic AD, we previously reported a nonlinear association pattern between brain volumes and CSF biomarker levels in some brain regions [55]. In this previous study, the parahippocampus and some parietotemporal regions presented an initial increase in volume in the low to intermediate biomarker abnormality level that later reverted. In this context, we hypothesize that the observed increase in GM volumes found in the SCD– group presented here might be capturing this initial effect of AD pathology that may be present in some subjects. Within the SCD group, on the contrary, the SCDplus classification criteria showed the capacity to detect individuals who, on average, displayed brain morphological differences associated with preclinical AD stages. In this regard, note that our sample was recruited from the general population rather than from memory clinics as in most SCD studies found in the literature. Therefore, the particular recruitment strategy in our study may underlie this unexpected finding which deserves further investigation. It is known that the recruitment strategy could highly affect SCD characteristics. Perrotin et al. [28] found differences between SCD subjects recruited from a memory clinic and those from the community. While both groups showed increased β-amyloid deposition and anxiety, subclinical depression and brain atrophy was found only in the SCD subjects who sought medical advice. Using VBM they reported brain atrophy in similar areas to those we found in our study in this latter group, suggesting that the presence of worry of enough intensity to induce help-seeking behavior would be a relevant feature of increased likelihood for AD. Similarly, Abdelnour et al. [29] reported worse neuropsychological performance in individuals with SCD attending a memory clinic than those recruited as study participants from the community, which may reflect a higher enrichment of AD in this group. Help-seeking has been recently suggested to occur concurrent with NIA–AA preclinical stage 2 (i.e., evidence of positive amyloid and tau markers) and to be strongly related to future cognitive decline [17, 30]. In the whole picture, our findings in the ALFA population-based cohort may reflect changes at an earlier stage to help-seeking behavior that can be captured at the group level by neuroimaging and challenging cognitive assessments.

This study is not free of limitations. The most important is the absence of biomarker data to be used as proxies of AD pathology. Ongoing studies in the follow-up of a subsample will solve this and will enable a refined analysis to further assess the usefulness of using the number of SCDplus features as a proxy of a continuum within SCD. In addition, as already highlighted throughout this paper, the value of each individual SCDplus feature seems to have a different weight in the prediction of cognitive performance. Ideally, homogeneous subgroups of subjects fulfilling each of the possible feature combinations would aid to disentangle interactions and reveal combinations of features of special interest. However, the sample size and the frequency of SCDplus features in the current study prevented us from using this approach.

## Conclusions

This study provides evidence of distinct cognitive and neuroimaging correlates of SCDplus subjects. Our analysis revealed complex interplays between cognitive performance and brain morphometry profiles that deserve further investigation. This approach may contribute to developing strategies to stratify SCD subjects at different risk levels of AD.

## Additional file

Additional file 1: **Table S1.** Sociodemographic, genetic, and mood descriptive data in the MRI sample. **Table S2.** Frequency of co-occurrence of SCDplus features in subjects with SCD in the whole sample (*n* = 572). **Table S3.** Frequency of co-occurrence of SCDplus features in subjects with SCD in the MRI subsample (*n* = 109). **Table S4.** Brain regions showing statistically significant negative associations between the number of SCDplus features met and GM volume. **Figure S1.** Frequency of SCD subjects by number of criteria met in the whole sample. **Figure S2.** Frequency of SCD subjects by number of criteria met in the MRI subsample. **Figure S3.** Brain regions showing a negative linear relation between number of SCDplus features met and GM volume in SCD subjects. (DOCX 441 kb)

## Abbreviations

AD: Alzheimer’s disease
APOE: Apolipoprotein E
CDR: Clinical Dementia Rating
CSF: Cerebrospinal fluid
FCSRT: Free and Cued Selective Reminding Test
FWHM: Full-width at half maximum
GADS: Goldberg Anxiety and Depression Scale
GM: Gray matter
MBT: Memory Binding Test
MCI: Mild cognitive impairment
MIS: Memory Impairment Screen
MMSE: Mini-Mental State Examination
MNI: Montreal Neurological Institute
MRI: Magnetic resonance imaging
PACC: Preclinical Alzheimer cognitive composite
SCD: Subjective cognitive decline
SCD-Q: Subjective Cognitive Decline Questionnaire
TDFR: Total delayed free recall
TDPR: Total delayed paired recall
TFR: Total free recall
TIV: Total intracraneal volume
TPR: Total paired recall
VBM: Voxel-based morphometry
WAIS: Wechsler Adult Intelligence Scale
WM: White matter
