# Supplementary material for: Brain and cognitive correlates of subjective cognitive decline-plus features in a population-based cohort
Source: Alzheimers Res Ther. 2018 Dec 20;10:123. doi: 10.1186/s13195-018-0449-9 (PMC6302483; doi:10.1186/s13195-018-0449-9)

**Table S1.** Sociodemographic, genetic and mood descriptive data in the MRI sample

|  | **Non-SCD** | **SCD-** | **SCD+** |
| --- | --- | --- | --- |
| N | 423 | 37 | 72 |
| Age M (SD) | 56.91 (7.30) | 56.67 (6.69) | 62.04 (7.12)^ab^ |
| Education M (SD) | 13.76 (3.55) | 14.14 (3.75) | 12.69 (3.42) ^a^ |
| Females n (%) | 250 (59.1%) | 23 (62.2%) | 46 (63.9%) |
| *APOE-ε4* Carriers n (%) | 216 (51.1%) | 9(24.3%) ^a^ | 47 (65.3%) ^ab^ |
| GADS- Anxiety M (SD) | .48 (1.07) | 1.00 (1.80) ^a^ | .93 (1.52) ^a^ |
| GADS- Depression M (SD) | .14 (.58) | .49 (1.04) ^a^ | .49 (1.01) ^a^ |

^a^p<.05 as compared to Non-SCD; pairwise (HSD Tukey) ANOVA/Chi-square test.

^b^p<.05 as compared to SCD-; pairwise (HSD Tukey) ANOVA/Chi-square test.

SCD-, SCD with 3 or less SCDplus features; SCD+, SCD with more than 3 SCDplus features; GADS, Goldberg Anxiety and Depression Scale.

**Table S2.** Frequency of co-occurrence of SCDplus features in subjects with SCD in the whole sample (n=572)

|  | Memory Complaint | Decline last 2y | Age over 60 | Worry | Confirmation by Inf. | E4 Carriers |
| --- | --- | --- | --- | --- | --- | --- |
| Memory Complaint | * |  |  |  |  |  |
| Decline last 2y | 412 | * |  |  |  |  |
| Age over 60 | 195 | 146 | * |  |  |  |
| Worry | 388 | 299 | 122 | * |  |  |
| Confirmation by Inf. | 159 | 118 | 60 | 116 | * |  |
| E4 Carriers | 196 | 145 | 60 | 139 | 57 | * |

**Table S3.** Frequency of co-occurrence of SCDplus features in subjects with SCD in the MRI subsample (n=109)

|  | Memory Complaint | Decline last 2y | Age over 60 | Worry | Confirmation by Inf. | E4 Carriers |
| --- | --- | --- | --- | --- | --- | --- |
| Memory Complaint | * |  |  |  |  |  |
| Decline last 2y | 85 | * |  |  |  |  |
| Age over 60 | 53 | 43 | * |  |  |  |
| Worry | 83 | 66 | 42 | * |  |  |
| Confirmation by Inf. | 39 | 31 | 21 | 31 | * |  |
| E4 Carriers | 56 | 46 | 28 | 43 | 19 | * |

**Table S4.** Brain regions showing statistically significant negative associations between the number of SCDplus features met and GM volume

|  | Label | MNI coordinates | | | Cluster | Peak | pFWE-corr | |
| --- | --- | --- | --- | --- | --- | --- | --- | --- |
|  |  | x | y | z | size | Z-score | peak | cluster |
| Cluster 1 | Left Temporal Inferior | -36 | -23 | -38 | 1744 | 4.87 | 0.007 | 0.025 |
|  | Left Fusiform |  |  |  |  |  |  |  |
|  | Left Temporal Pole Superior |  |  |  |  |  |  |  |
|  | Left Temporal Superior |  |  |  |  |  |  |  |
| Cluster 2 | Left Temporal Superior | -60 | -35 | 14 | 2043 | 4.33 | 0.061 | 0.015 |
|  | Left Temporal Middle |  |  |  |  |  |  |  |
|  | Left Temporal Inferior |  |  |  |  |  |  |  |
| Cluster 3 | Right Temporal Superior | 71 | -30 | 0 | 217 | 4.31 | 0.066 | 0.622 |
|  | Right Temporal Middle |  |  |  |  |  |  |  |
| Cluster 4 | Right Frontal Superior | 24 | 27 | 42 | 544 | 4.25 | 0.084 | 0.295 |
|  | Right Frontal Middle |  |  |  |  |  |  |  |
| Cluster 5 | Right Frontal Inferior Operc. | 51 | 8 | 18 | 592 | 4.13 | 0.127 | 0.264 |
|  | Right Rolandic Operculum |  |  |  |  |  |  |  |
|  | Right Frontal Inferior Triang. |  |  |  |  |  |  |  |
| Cluster 6 | Vermis | -2 | -57 | -14 | 3885 | 4.00 | 0.194 | 0.001 |
|  | Left Precuneus |  |  |  |  |  |  |  |
|  | Left Cerebellum |  |  |  |  |  |  |  |
|  | Left Cuneus  Right Cerebellum |  |  |  |  |  |  |  |
| Cluster 7 | Right Rolandic Operculum | 50 | -24 | 32 | 2724 | 3.96 | 0.219 | 0.005 |
|  | Right Postcentral |  |  |  |  |  |  |  |
|  | Right Temporal Superior |  |  |  |  |  |  |  |
|  | Right Supramarginal |  |  |  |  |  |  |  |
|  | Right Insula |  |  |  |  |  |  |  |
|  | Right Heschl |  |  |  |  |  |  |  |
| Cluster 8 | Left Postcentral | -51 | -21 | 30 | 469 | 3.87 | 0.282 | 0.350 |
|  | Left Supramarginal |  |  |  |  |  |  |  |
|  | Left Parietal Inferior |  |  |  |  |  |  |  |
| Cluster 9 | Right Frontal Superior Medial | 3 | 48 | 32 | 1286 | 3.77 | 0.372 | 0.060 |
|  | Left Frontal Superior Medial |  |  |  |  |  |  |  |
|  | Right Frontal Superior |  |  |  |  |  |  |  |
|  | Right Cingulate Anterior |  |  |  |  |  |  |  |
| Cluster 10 | Right Cerebellum Crus | 35 | -74 | -27 | 1150 | 3.72 | 0.426 | 0.079 |
|  | Right Cerebellum |  |  |  |  |  |  |  |
| Cluster 11 | Right Fusiform | 50 | -24 | -20 | 777 | 3.70 | 0.441 | 0.174 |
|  | Right Temporal Inferior |  |  |  |  |  |  |  |
| Cluster 12 | Left Cerebellum | -11 | -63 | -48 | 955 | 3.59 | 0.563 | 0.119 |
|  | Right Cerebellum |  |  |  |  |  |  |  |
|  | Vermis |  |  |  |  |  |  |  |
| Cluster 13 | Right Insula | 36 | 24 | -6 | 153 | 3.52 | 0.637 | 0.714 |
|  | Right Frontal Inferior Orb. |  |  |  |  |  |  |  |
| Cluster 14 | Left Rectus | -8 | 41 | -18 | 439 | 3.49 | 0.664 | 0.375 |
|  | Left Frontal Medial Orb. |  |  |  |  |  |  |  |

MNI: Montreal Neurological Institute; only labels accounting for more than 5% of the cluster volume are reported

**Figure S1.** Frequency of SCD subjects by number of criteria met in the whole sample


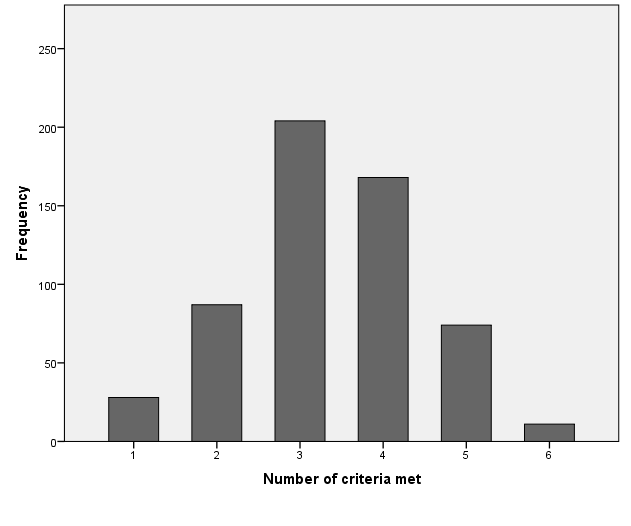


**Figure S2.** Frequency of SCD subjects by number of criteria met in the MRI subsample


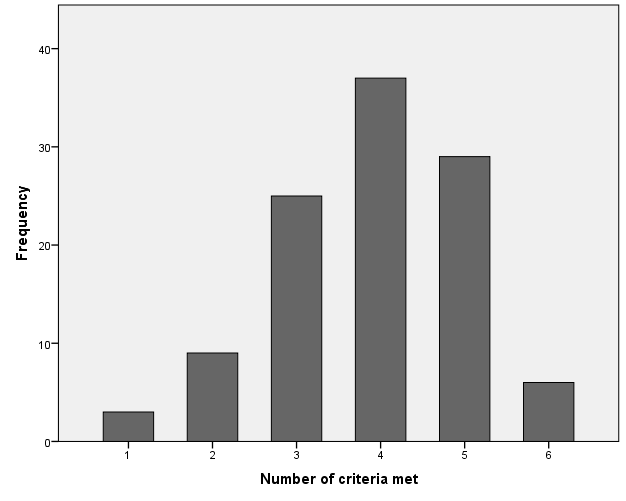


**Figure S3.** Brain regions showing a negative linear relation between number of SCDplus features met and GM volume in SCD subjects


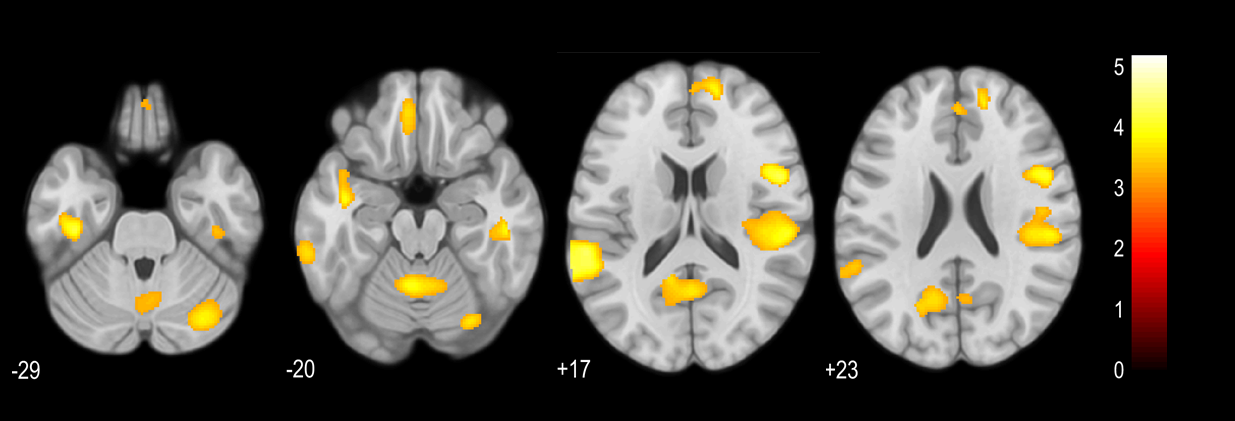

Supplement: Supplementary file 1 — Table S1. Sociodemographic, genetic, and mood descriptive data in the MRI sample. Table S2. Frequency of co-occurrence of SCDplus features in subjects with SCD in the whole sample (n = 572). Table S3. Frequency of co-occurrence of SCDplus features in subjects with SCD in the MRI subsample (n = 109). Table S4. Brain regions showing statistically significant negative associations between the number of SCDplus features met and GM volume. Figure S1. Frequency of SCD subjects by number of criteria met in the whole sample. Figure S2. Frequency of SCD subjects by number of criteria met in the MRI subsample. Figure S3. Brain regions showing a negative linear relation between number of SCDplus features met and GM volume in SCD subjects. (DOCX 441 kb) [file 13195_2018_449_MOESM1_ESM.docx]
